# Mechanistic insights into promotion of non-small cell lung cancer by BAG5 using integrative multi-omics approaches

**DOI:** 10.3389/fimmu.2025.1648139

**Published:** 2025-07-25

**Authors:** Jing-Shan Huang, Jia-Mei Wang, Ye Yuan, Ting Zhang, Bai-Qiang Li, Fu-Ying Zhao, Liang Hao, Zhan-Wu Yu, Hua-Qin Wang

**Affiliations:** ^1^ Department of Thoracic Surgery, the Shengjing Hospital, China Medical University, Shenyang, China; ^2^ Department of Biochemistry & Molecular Biology, China Medical University, Shenyang, China; ^3^ Innovative Cancer Drug Research and Development Engineering Centre of Liaoning Province, The Shengjing Hospital, China Medical University, Shenyang, China; ^4^ Department of Laboratory Medicine, The 1st Affiliated Hospital, China Medical University, Shenyang, China; ^5^ Department of Chemistry, School of Forensic Medicine, China Medical University, Shenyang, China; ^6^ Department of Thoracic Surgery, Cancer Hospital of China Medical University, Liaoning Cancer Hospital & Institute, Shenyang, China

**Keywords:** BAG5, NSCLC, multi-omics, metabolic reprogramming, EMT

## Abstract

**Introduction:**

With the continuous emergence of new technologies in omics, the integrative analysis of multi-omics data has become a new direction to explore life mechanisms. The Bcl-2 associated athanogene (BAG) family consists of co-chaperones involved in various cellular processes, including stress signaling, cell cycle regulation, and tumorigenesis. BAG5, a unique member of this family, contains multiple BAG domains, yet its role in non-small cell lung cancer (NSCLC) remains largely unexplored.

**Methods:**

In this study, we employed a multi-omics approach, integrating single-cell transcriptomics, proteomics, interactomics, and phosphoproteomics data to comprehensively investigate BAG5 function in NSCLC. Functional analyses were performed using cell lines and patient-derived organoids (PDOs) to validate our findings.

**Results:**

Our results demonstrate that BAG5 plays a critical role in the regulation of RNA metabolism, mitochondrial dynamics, and metabolic reprogramming. Additionally, BAG5 is involved in cytoskeletal remodeling and epithelial-to-mesenchymal transition (EMT), contributing to the proliferation and invasion of NSCLC cells.

**Discussion:**

These findings underscore the potential oncogenic role of BAG5 in NSCLC, revealing that it acts through multiple molecular pathways. Our study suggests that targeting BAG5 could be a promising therapeutic strategy for treating NSCLC.

## 1 Introduction

Lung cancer is one of the most frequent malignancies and a leading cause of cancer-related deaths (1). Non-small cell lung cancer (NSCLC) represents approximately 85% of all lung cancer cases (2). The traditional treatment methods include operation, chemotherapy and radiotherapy, but for the patients with advanced lung cancer, the 5-year overall survival rate is less than 15% (2). An in-depth study of the mechanisms underlying the characteristics of NSCLC proliferation, migration and invasion may provide new therapeutic strategies for NSCLC patients.

The Bcl-2 associated anthanogene (BAG) family is a family of co-chaperones comprising of six members that play important roles in cell survival and death pathways (3). Each BAG family member (BAG1-6) contains at least one evolutionarily conserved BAG domain, which allows BAG protein to interact with the HSP70 family (4, 5). BAG proteins interact with a variety of proteins and is involved in a variety of cellular processes, such as stress signaling, cell cycle and tumorigenesis (3, 4, 6). BAG5 is an unique BAG family as it consists of five BAG domains in tandem and attenuates the activity of the chaperone HSP70 (6, 7). BAG5 is a regulator of Parkin-dependent mitophagy and cell death (8). BAG5 also interacts with DJ1 and prevents mitochondrial oxidative damage (9). BAG5 regulates endoplasmic reticulum (ER) stress and is implicated in the regulation of oxidative stress, apoptosis and inflammation (10). BAG5 might have both tumor-promoting and tumor-suppressive function in a cell-context dependent manner. BAG5 promotes proliferation and invasion of pancreatic cancer, as miR-127 functions as a tumor suppressor via directly targeting BAG5 (11). BAG5 functions as downstream of circ_0008305 to trigger hepatocellular carcinoma (HCC) progression (12). In addition, deficiency of PRMT6 promotes cell survival in hostile microenvironments of HCC tumors by regulating BAG5-associated HSC70 stability through post-translational methylation of BAG5 (13). BAG5 also promotes invasion of papillary thyroid cancer cells via promoting translation of fibronectin (14). On the contrary, BAG5 is identified to suppress the proliferation and invasion of MCF7 by stabilizing PTEN protein (15). It has been reported that BAG5 inhibits mutant p53 degradation and promotes gain-of-functions (GOF) of mutant p53 in tumorigenesis (16). BAG5 functions as a tumor promoting factor in p53 mutant (17, 18), while as a tumor suppressor in p53 wild type or deletion ovarian cancer cells (19), indicating that as least p53 status might be implicated in the paradoxical function of BAG5 in tumor progression.

With the continuous emergence of new technologies in omics, the development of omics research in the direction of quantification and high throughput has been accelerated. Through the integrative analysis of multi-omics data, it has become a new direction for scientists to explore life mechanisms. In the current study, we examined the biological processes by which BAG5 regulated growth and invasion of NSCLC using multi-omics approach integrating single cell transcriptomics, proteomics, interactomics and phosphoproteomics data from parental and BAG5 knock-out cells and patient-derived organoids (PDO) to identify function of BAG5 in NSCLS.

Despite prior studies suggesting context-dependent roles for BAG5 in tumor biology, its specific functions and regulatory mechanisms in NSCLC remain largely undefined. To our knowledge, this study is the first to comprehensively investigate BAG5 in NSCLC using an integrative multi-omics approach. By combining single-cell transcriptomics, proteomics, phosphoproteomics, and functional validation in PDO/PDX models, we bridge the knowledge gap surrounding BAG5-mediated tumor pathways in NSCLC and provide novel insights with translational implications.

## 2 Materials and methods

### 2.1 Cell culture

NSCLC cells (A549, HCC817, PC9, H1299, H1975, SK-MES-1), the normal human lung epithelial cell line BEAS-2B and 16HBE were obtained from ATCC. All cells were verified through short tandem repeat profiling. Cells were maintained in RPMI 1640 medium (Biological Industries, Israel) supplemented with 10% fetal bovine serum (FBS, Gibco, USA) and 1% penicillin-streptomycin (Sigma) in a humidified atmosphere of 5% CO2 at 37 °C. All cells were routinely tested and found negative for mycoplasma.

### 2.2 Lentiviral infection

Lentiviral-based CRISPR/Cas9 gene editing system (lentiCRISPR) was used to establish BAG5 knockout NSCLC cells and PDO. Single guide RNAs (sgRNAs) targeting BAG5 were cloned into lentiCRISPR vector. NSCLC cells and PDO were transduced with lentiCRISPR-BAG5 and selected with puromycin. The empty sgRNA vector served as a control. The sgRNA sequence targeting BAG5 was listed as follows: BAG5 sgRNA#1, 5’-ACCAACATCCTTCTATTAGT-3’; BAG5 sgRNA#2, 5’-TCTGTATTTCAATCCGGTGT-3’; BAG5 sgRNA#3, 5’-GTGATACCTTGCTTTCCGCA-3’. Identification of knockout was carried out by western blot.

### 2.3 Human tissue samples

31 paired NSCLC tissues and adjacent paratumor tissues were collected from patients during surgery at Shengjing Hospital of china Medical University. Tissue from patients was snap frozen in liquid nitrogen and stored in −80°C for further immunoblot analysis. None of the patients had received chemotherapy or radiotherapy prior to the operation. This study was approved by the ethics committee on Human Research of Shengjing Hospital of china Medical University and written informed consent was obtained from all parents/guardians.

### 2.4 Patient-derived organoid

Patient-derived organoid was generated as previously described (20, 21). Unlike PDX models, organoids were cultured and analyzed exclusively *in vitro* for functional validation, without implantation into nude mice. Briefly, NSCLC tissues from patients was minced into small pieces (1–3 mm^3^) and digested by collagenase II and TrypLE Express Enzyme at 37 °C. Organoids were then embedded within Matrigel and cultured in 24-well plates supplemented with human complete feeding medium in a humidified incubator with 5% CO2 at 37 °C.

### 2.5 Co-immunoprecipitation and interactome of BAG5

Cells were harvested and lysed by NP-40 lysis buffer (50 mM Tris-Hcl pH 7.4, 150 mM NaCl) supplemented with complete protease inhibitors (Sigma) on ice for 30 min. Cell lysate was centrifuged for 20 min at 12,000 × g at 4 °C. Co-IP for exogenous expressed proteins, supernatant was incubated with anti-Flag magnetic beads (Bimake). After incubation overnight at 4°, beads were washed four times with lysis buffer. The antigen-antibody-magnetic bead complex was mixed with 60 μL 1 × SDS-PAGE loading buffer and heated at 100°C for 10 min. After the magnetic beads were adsorbed on the bottom of the centrifuge tube, the supernatant was collected for further SDS-PAGE detection. Coomassie brilliant blue staining was applied to reveal potential distinct bands in BAG5 containing immunoprecipitation complex from NSCLC cell lines compared to immortalized lung epithelial cell lines. The antigen-antibody-magnetic bead complex was obtained as described above. Then the LC-MS/MS analysis was conducted to evaluate the isolated immunoprecipitates (PTM biolabs, Hangzhou, China). Mass spectrometry data is provided in **Supplementary Table S1**.

**Figure 1:**
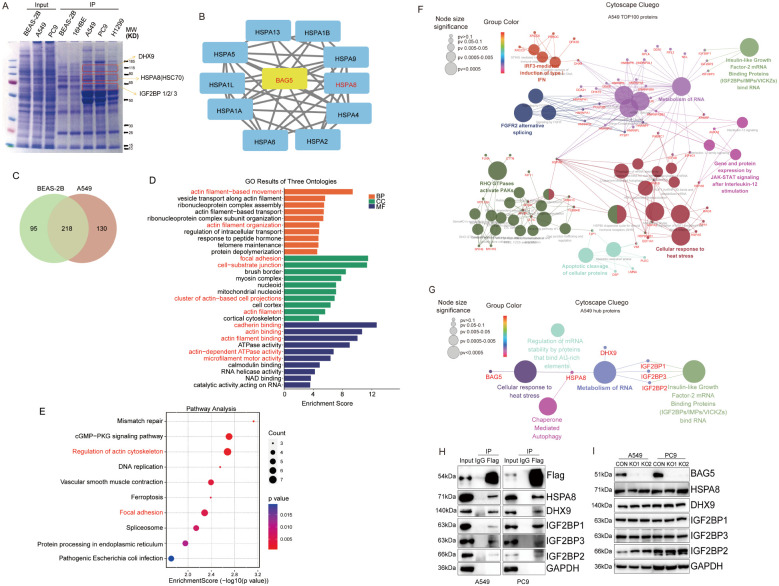
Involvement of BAG5 interactome in regulation of actin cytoskeleton, focal adhesion and RNA metabolism. **(A)** Coomassie blue staining of SDS-PAGE gel analyzed BAG5 immunocomplex from a panel of immortalized lung epithelial and NSCLC cell lines. **(B)** SDS-PAGE gel from lung epithelial cell line BEAS-2B and NSCLC cell line A549 were identified by mass spectrometry. Ten members of Hsp70/HSC70 family were identified to interact with BAG5 in A549 cell. **(C)** Venn diagram showed the different interacting proteins between BEAS-2B and A549 cell. **(D, E)** GO **(D)** and KEGG pathways **(E)** enrichment analysis of 130 unique BAG5-interacting proteins in A549 cell. **(F, G)** Cytoscape ClueGO visualizing the enriched pathways of TOP 100 **(F)** and TOP 5 **(G)** hub proteins unique in A549 cell. **(H)** Co-IP was performed to confirm the potential interaction of BAG5 with the top 5 of BAG5-interacting proteins influencing protein translation. **(I)** BAG5-interacting proteins were analyzed by western blot analysis.

Approximate molecular weights of selected interactors (e.g., DHX9 ~140 kDa, HSPA8 ~70 kDa, IGF2BPs ~65–70 kDa) were used as a visual reference in **Figure 1A**. The labeling in the figure corresponds to regions where these proteins were most abundantly detected by MS.

### 2.6 Proteomics and phosphorproteomics and data analysis

Total proteins from A549 cells infected with control sgRNA and BAG5-sgRNA lentiviral constructs were extracted and subjected to label-free quantitative proteomics and phosphoproteomics analysis using LC-MS/MS. Protein lysates were digested with trypsin, and peptides were desalted prior to analysis. For phosphoproteomic profiling, phosphopeptides were enriched using TiO_2_-based affinity chromatography. Samples were then analyzed on a high-resolution Orbitrap mass spectrometer (Thermo Scientific) coupled with a nanoLC system.

Raw data were processed using MaxQuant software with the Andromeda search engine, and protein identification was performed against the UniProt human database. Label-free quantification (LFQ) intensity values were used for comparative analysis. Phosphorylation site localization probability ≥ 0.75 (as determined by MaxQuant). and fold change ≥ 1.5 or ≤ 0.67, with a p-value < 0.05, were set as screening criteria for significant differential phosphorylation events. Functional enrichment and kinase-substrate network analysis were performed using DAVID and KSEA tools.

The LC-MS/MS and bioinformatics of DEGs (1.5<Fold change or Fold change<1/1.5, and adjusted *P*-values < 0.05) were performed by PTM biolabs, Hangzhou, China. Mass spectrometry data is provided in **Supplementary Tables S2** and **S3**, respectively.

### 2.7 Functional enrichment analysis

Gene Ontology (GO) and Kyoto Encyclopedia of Genes and Genomes (KEGG) of DEGs were analyzed utilizing clusterProfiler package. Furthermore, BAG5 interacting proteins were analysed using ClueGO v2.5.8 on Cytoscape version 3.9.0 for Gene Ontology biological function and KEGG pathway identification by applying a two-sided hypergeometric test with a *p* value cut-off of <0.05. The hallmark gene sets were acquired from the Molecular Signatures Database (http://software.broad-institute.org/gsea/msigdb). Gene set variation analysis (GSVA) was adopted to ascribe signaling pathway variation scores to the gene sets, thus assessing the biological significance.

### 2.8 TCGA and GTEx datasets processing

RNA-sequencing expression profiles and corresponding clinical information for BAG5 were downloaded from the TCGA dataset (https://portal.gdc.com). The current-release (V8) GTEx datasets were obtained from the GTEx data portal website (https://www.gtexportal.org/home/datasets). Statistical analyses were performed using R software v4.0.3 (R Foundation for Statistical Computing, Vienna, Austria). *P*-value <0.05 was considered statistically significant.

### 2.9 Single-cell sequencing data

The scRNA-seq count matrix of 20 primary non-small cell lung cancer (NSCLC) patients diagnosed with stage I-IV was downloaded from the GSE119911 cohort (https://www.ncbi.nlm.nih.gov/geo/). Quality control was implemented with Seurat package. Single cells with > 20% mitochondrial UMI counts as low-quality cells were removed. Batch effects were eliminated utilizing Integrate Data function. The top 20 principal components (PCs), and the top 2000 highly variable genes, were selected. Influence of the percentage of mitochondrial UMI counts were removed with ScaleData function. Afterwards, cell populations were clustered with FindClusters function, and visualized with t-distributed stochastic neighbor embedding (t-SNE). The markers of each cell cluster were determined with FindAllMarkers function. The main cell types were determined on the basis of markers acquired from the CellMarker database (http://biocc.hrbmu.edu.cn/CellMarker/).

### 2.10 Gene set variation analysis and functional annotation

To investigate the difference on Gene Ontology (GO) and pathways between BAG5^+^ and BAG5^-^ tumor epithelial cells, GSVA enrichment analysis was performed using “GSVA 1.46.0” R packages. The gene sets of “ C2, C5, H” for “GO, KEGG, HALLMARK” were downloaded from MSigDB database for running GSVA analysis. Adjusted *P* with value less than 0.05 was considered as statistically significance.

### 2.11 Immunoblot analysis

Total cell lysates or immunoprecipitates prepared as described above were separated by SDS-PAGE, transferred onto PVDF membranes (Millipore, USA), blocked in 5% nonfat milk and then incubated with primary antibodies overnight at 4 °C and corresponding secondary antibodies for 2 h at room temperature, and visualized by ECL western blotting detection reagent (Pierce). The primary antibodies used in the current study were listed as following: BAG5 (Sigma, USA), HK2 (Cell Signaling Technology, USA), HSPA8, IGF2BP1, IGF2BP3, E-cadherin, N-cadherin, ZEB1 and Flag (Cell Signaling Technology, USA), β-actin (Immunoway, USA), DRP1 and MFN2 (Abcam, USA), GLUT3, DHX9, IGF2BP2 and β-catenin (Santa Cruz Biotechnology, USA), and GAPDH (Millipore, USA).

### 2.12 H&E staining and immunohistochemistry

The hematoxylin and eosin (H&E) (Beyotime Biotechnology) was applied to stain for nuclei and cytoplasm respectively according to manufacturer’s instructions. For immunohistochemistry, tissues were incubated with 4% paraformaldehyde, embedded in paraffin, and then sliced into slices. After primary antibody against BAG5 (Sigma, USA) or Ki67(Abcam, USA) and secondary antibody incubations, HRP-labelled streptavidin solution was added to the samples for 15 min. The immunocomplex was visualized with DAB, and the nuclei were counterstained with haematoxylin.

### 2.13 Colony formation assay

For the plate colony formation assay, 200 cells were seeded into each 6-well plate (Corning, USA) and cultured in a humidified incubator at 37°C with 5% CO2. After 14 days, when there were visible colonies in the plate, cells were fixed with 4% paraformaldehyde for 15 min and then stained with 0.1% crystal violet for 30 min. The number of valid colonies (those containing more than 50 cells) were calculated using ImageJ software (NIH, USA).

### 2.14 Transwell migration and invasion assays

Migration and invasion assays were performed using Transwell chamber system (Corning, USA). For migration assay, 3 × 10^4^ cells were seeded in the upper chamber of an insert with 0.1 ml FBS-free starvation medium, and 0.6 ml culture media with 10% FBS were added outside the chamber in the wells of the plate. For invasion assays, the upper chamber of the insert was pre-coated with Matrigel (Millipore Sigma) before plating cells. After incubation for 48 h, cells were fixed with 4% paraformaldehyde for 1 hour and then stained with 0.1% crystal violet for 30 min. After rinsing with water and airing, migrating or invading cells were imaged and counted using a Cytation 5 microscope (BioTek, USA).

### 2.15 Spheroid formation assay

Cells were digested with 0.25% trypsin, centrifuged at 800 rpm for 5 min, and resuspended in serum-free DMEM/F12 medium (Biological Industries, Israel). Cells (4 × 10^4^) were seeded into ultralow-attachment 6-well plates (Corning, USA) and cultured in 3 ml of serum-free DMEM/F12 medium supplemented with 20 mg/ml human recombinant epidermal growth factor, 5 μg/mL insulin (both from Sigma-Aldrich) and 2% B27 (Invitrogen). During the culture, the medium was replaced every 3 days. After 10–14 days, cells were photographed using a Cytation 5 Cell Imaging Multi-Mode Reader (BioTek Instruments, USA). Spheroids exceeding 50 μm in diameter were counted.

### 2.16 Seahorse metabolic analysis

ECAR and OCR were measured using Seahorse XF Glycolysis Stress Test Kit and Seahorse XF Cell Mito Stress Test Kit (Agilent Technologies, Palo Alto, CA), respectively. NSCLC cells were seeded into the 96-well cell culture plates in medium with 10% FBS and incubated at 37°C overnight and then the cells were used for measurement of ECAR and OCR. After measurement of baseline concentration, glucose, oligomycin, and 2-DG were sequentially added into each well for ECAR measurement. Oligomycin, FCCP (p-trifluoromethoxy carbonyl cyanide phenylhydrazone), and Antimycin A & Rotenone were sequentially injected into each well for OCR measurement. Seahorse XF-96 Wave software was used to analyze the data.

### 2.17 HPG incorporation assay

Cells were incubated with L-homopropargylglycine (HPG) for 30 min, followed by click-iT labeling and fluorescence quantification to assess nascent protein synthesis.

### 2.18 2-NBDG uptake assay

2-NBDG was added to live cells at 100 μM for 30 minutes, then washed and measured by flow cytometry for glucose uptake.

### 2.19 Mitochondrial staining

Cells were incubated with MitoTracker Red CMXRos (50 nM) for 30 minutes at 37°C, followed by fixation and confocal microscopy.

### 2.20 Flow cytometry for ROS

Intracellular ROS levels were measured using DCFH-DA probe (10 μM) incubated for 30 minutes before flow cytometry analysis.

### 2.21 Confocal immunofluorescence imaging

NSCLC cells were fixed with 4% paraformaldehyde for 15 minutes at room temperature, permeabilized using 0.1% Triton X-100 in PBS for 10 minutes, and blocked with 5% BSA for 1 hour. Primary antibodies were incubated overnight at 4°C, followed by appropriate fluorescently labeled secondary antibodies for 1 hour at room temperature. Nuclei were counterstained with DAPI (1 μg/mL, 5 minutes). Confocal images were acquired using a Leica TCS SP8 confocal laser scanning microscope with a 63× oil-immersion objective. Laser lines at 405, 488, and 561 nm were used for excitation, and emission bandwidths were adjusted accordingly. Images were processed using Leica LAS X and ImageJ software.

### 2.22 Transmission electron microscopy

Cells were fixed in 2.5% glutaraldehyde in 0.1 M phosphate buffer (pH 7.4) for 2 hours at 4°C, post-fixed in 1% osmium tetroxide for 1 hour, dehydrated through a graded ethanol series, and embedded in epoxy resin. Ultrathin sections (70 nm) were cut using a Leica EM UC7 ultramicrotome and stained with uranyl acetate and lead citrate. Sections were examined using a JEOL JEM-1400 Plus transmission electron microscope operating at 80 kV. Images were captured using a Gatan Orius CCD camera.

### 2.23 Mitochondrial morphology analysis

Cells were stained with mito-tracker. Z-stack confocal images were segmented in FIJI/ImageJ, and individual mitochondria were classified into three categories — fragmented (length ≤1 µm and circularity ≥0.8), intermediate (length 1–4 µm, no branching), and elongated (length >4 µm and/or ≥1 branch point) — following criteria adapted from Ong SB et al. (22) and L.-C. Tábara et al. (23). At least 100 mitochondria per condition from ≥10 cells were scored by two blinded observers.”

### 2.24 *In vivo* xenograft model

BALB/c-nu/nu mice (4–5 weeks old, female) were purchased from Liaoning Changsheng Biotechnology Co., Ltd. All animal procedures were approved by and compiled with the guidelines of the Institutional Animal Care Committee of China Medical University. Mice were subcutaneously inoculated with the specified number of viable A549 and PC9 cells. The status of the mice and tumor formation were observed over time, and the mice were sacrificed after 28 days. The subcutaneous tumors were removed and photographed.

### 2.25 *In vivo* metastasis assay

Five-week-old male BALB/c nude mice were maintained and handled according to instructions approved by the Animal Care Committee of China MedicaUniversity. The indicated stably transfected A549 cells (5 × 10^6^/0.1 ml PBS) were tail-vein injected into the nude mice. All mice were sacrificed 80 days later, and the lungs were surgically dissected. The lung tissues were embedded in paraffin for hematoxylin and eosin (H&E) staining and statistical analysis of the number of tumor nodules.

### 2.26 Statistical analyses

Experimental data are expressed as mean ± standard error and were analyzed using SPSS 23.0 (IBM, USA). According to the normality and variance homogeneity of the data, one-way analysis of variance (one-way ANOVA) and Dunnett’s *post hoc* test were adopted to analyze the differences among groups. Student’s t-test was conducted to analyze the differences in gene expression. Additionally, the log-rank test was used to compare overall survival distribution, chi-square (χ2) test was used to compare the difference of composition ratio, and Pearson’s correlation test was used to calculate the coefficient of association. *In vitro* experiments were repeated three times independently. A *P*-value <0.05 was considered statistically significant.

## 3 Results

### 3.1 BAG5 was highly expressed in tumor epithelial cells and correlated with NSCLC metastasis

**Figure 2:**
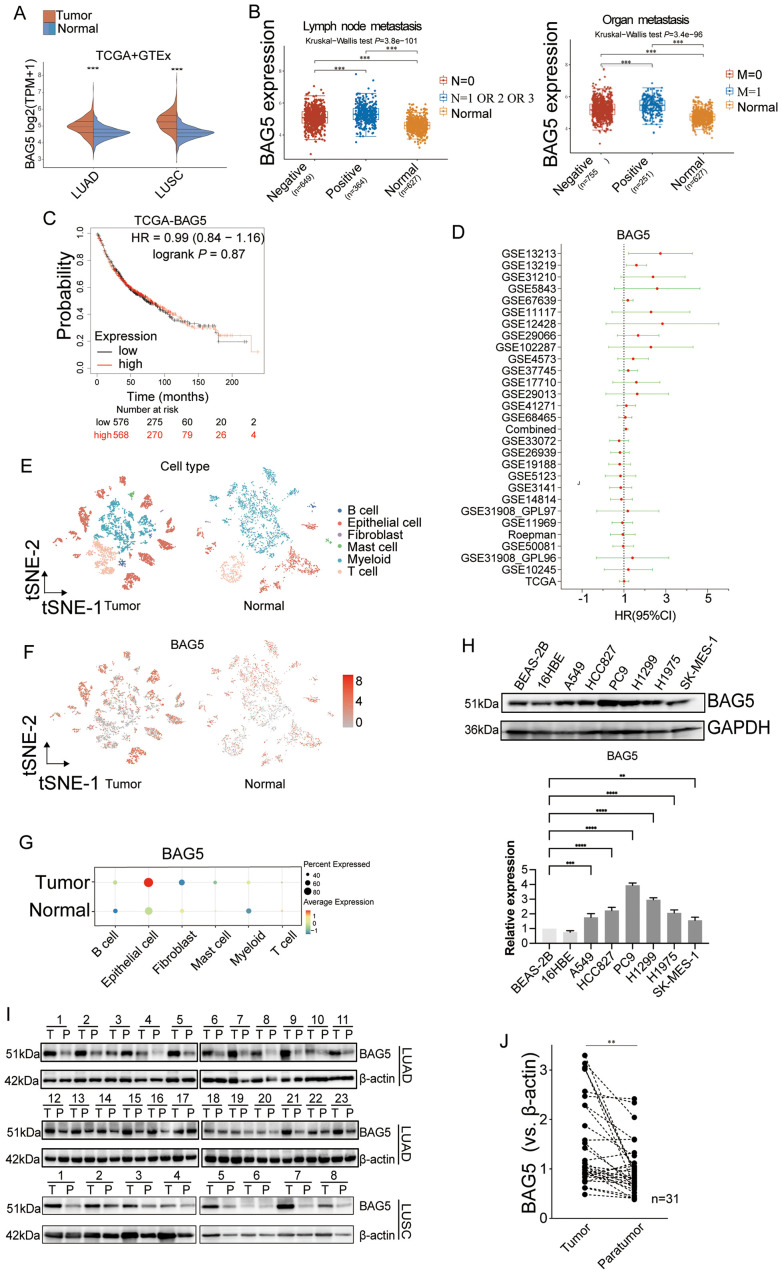
BAG5 was highly expressed in tumor epithelial cells and correlated with NSCLC metastasis. **(A, B)** Expression levels of BAG5 in tumor versus normal tissues were analyzed using a combined TCGA and GTEx dataset. Comparisons include lung adenocarcinoma (LUAD) and lung squamous cell carcinoma (LUSC), as well as subgroups with lymph node (N) or distant metastasis (M). ***p < 0.001; The statistical difference of two groups was compared through the Wilcox test, significance difference of three groups was tested with Kruskal-Wallis test. **(C)** Kaplan-Meier survival curve was constructed for NSCLC patients from TCGA based on BAG5 expression levels (BAG5-High vs. BAG5-Low). Significance (*p*) was evaluated by Log-rank test. HR: hazard ratio. **(D)** Forest plot of hazard ratios (HR) for the association of BAG5 expression with overall survival (OS) across 27 NSCLC datasets from the OSluca online database. Detailed statistics, including HR, 95% CI, and p-values for each dataset, are provided in Supplemental Table OSluca. **(E–G)** The public single-cell RNA-seq dataset (GSE119911) was reanalyzed. BAG5 expression levels across major cell types in NSCLC versus normal lung tissues are shown. In panel H, average expression levels of BAG5 are presented as a heatmap across annotated cell types. **(H)** Western blotting analysis was performed to examine BAG5 expression in a panel of normal and NSCLC cell lines, with GAPDH used as the loading control. Representative immunoblots are shown (upper), and densitometric quantification of relative BAG5 protein levels normalized to GAPDH is presented (lower). Statistical significance was evaluated using one-way ANOVA, ****p < 0.0001. **(I)** BAG5 protein level was investigated using Western blot in paired fresh NSCLC tumor (T) and paratumor (P) normal tissues, and representative images were provided. β-actin was used as a loading control. LUAD **(J)** Scatter plots showing relative expression of BAG5 in paired NSCLC tumor (T) and paratumor (P) normal tissues. **p < 0.01.

To investigate the expression landscape and clinical relevance of BAG5 in NSCLC, we first examined its transcriptional levels using the combined TCGA and GTEx datasets. BAG5 was significantly overexpressed in NSCLC tissues compared to normal controls (**Figure 2A**) and positively correlated with lymph node and distant organ metastasis (**Figure 2B**), which may affect the prognosis of patients. However, TCGA database showed that the expression level of BAG5 did not affect the prognosis of patients (HR = 0.99, P = 0.87) (**Figure 2C**). Further combining the GEO data set, using the online cancer integration for lung cancer (OSluca) (24), a multi-sample expansion analysis found that the prognostic impact of BAG5 on NSCLC fluctuated significantly, with HR fluctuating between 0.56 and 2.46 (**Figure 2D**, **Supplementary Table** OSsluca). These results suggest that tumor heterogeneity may obscure the prognostic value of BAG5 in bulk RNA sequencing datasets, underscoring the need for higher-resolution approaches such as single-cell or spatial transcriptomics in biomarker evaluation.

To address this limitation, the expression profile of BAG5 in the tumor microenvironment was then investigated at the single-cell level. Based on the canonical cell-type marker genes, cells from online NSCLC single-cell data sets were divided in 6 major cell types including B cells, epithelial cells, fibroblasts, mast cells, myeloid cells, and T cells (**Figure 2E**). While cells were classified into six major lineages for global comparison, we acknowledge the complexity of the tumor microenvironment and the potential for further subclassification, particularly within immune and stromal compartments. However, given that BAG5 expression was strongly enriched in tumor epithelial cells, downstream analyses focused on epithelial subclusters to investigate functional heterogeneity within this population. The expression level of BAG5 in tumor epithelial cells was significantly higher than that in other cell types, as well as than that in normal lung epithelial cells (**Figures 2F–G**), suggesting that BAG5 may be associated with malignant progression of NSCLC and may have a specific role in tumor epithelial cells.

BAG5 expression levels were relatively higher in a panel of NSCLC cell lines compared with human normal lung epithelial cell lines BEAS-2B and 16HBE (**Figure 2H**). Western blot analysis also showed that BAG5 expression was significantly higher in most of NSCLC tissues compared with paired para-cancer tissues (**Figures 2I, J**).

These results suggest that BAG5 is specifically overexpressed in NSCLC tumor epithelial cells and may be involved in disease progression and metastasis, although bulk RNA-seq may not sufficiently capture its prognostic relevance due to tumor heterogeneity.

### 3.2 BAG5 knockout inhibited proliferation and invasion of NSCLC *in vitro* and *in vivo*

**Figure 3:**
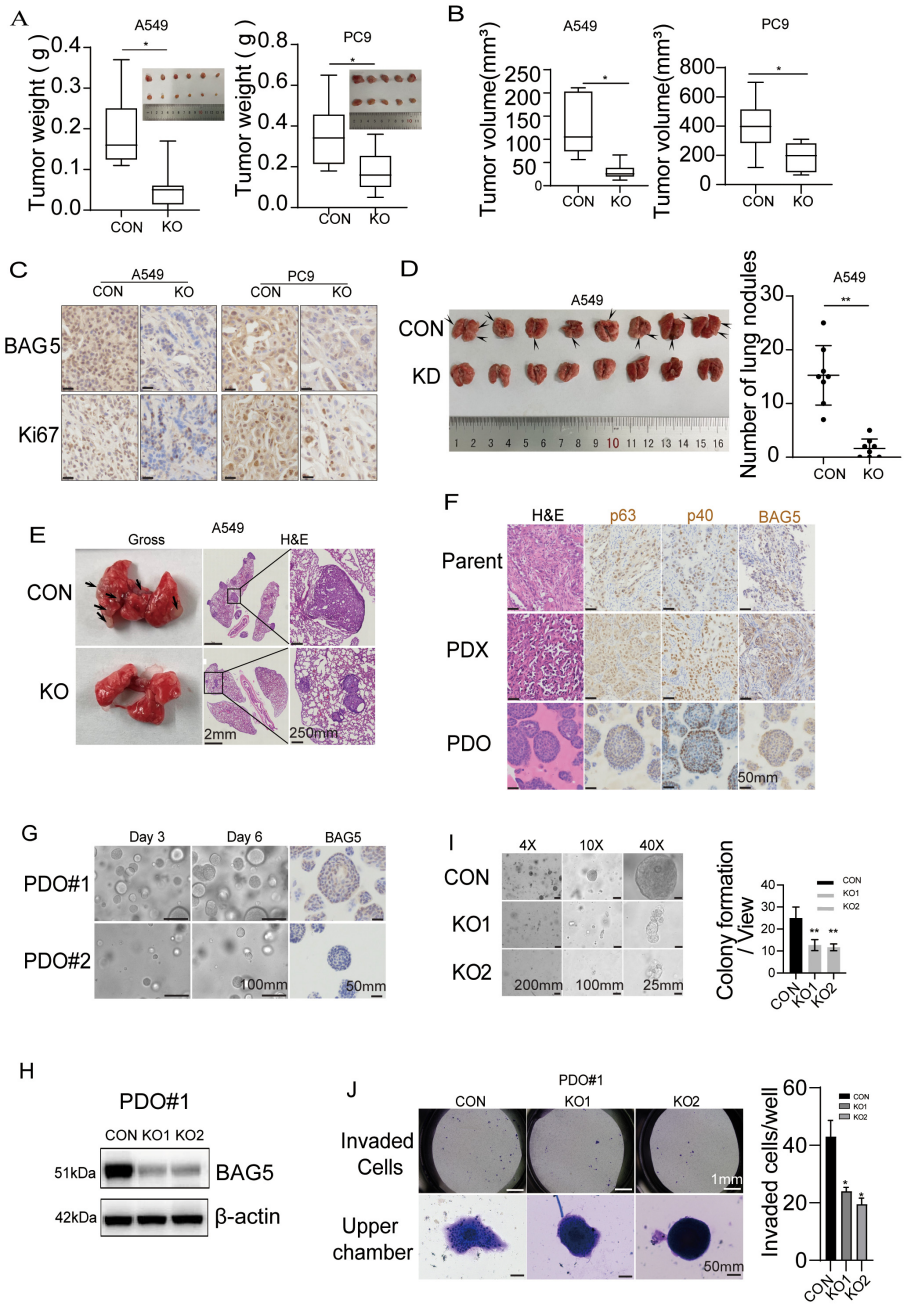
BAG5 knockout inhibited proliferation and invasion of NSCLC *in vitro and in vivo*. **(A, B)** Control and BAG5 knockout NSCLC cells were injected subcutaneously on the right flanks of nude mice (n = 5-6 mice per group). Tumors of each group were removed and photographed after sacrifice of animals. Tumor weight and volumes (mean ± SD) were analyzed. *p < 0.05. **(C)** Xenografts were sectioned and stained with BAG5 and Ki67. Scale bars, 25μm. **(D, E)** To evaluate spontaneous metastasis, BAG5 knockout and control A549 cells were i.v. injected to nude mice (n = 8 mice per group). Representative images (**D** left), quantified values (**D** right) and H&E staining **(E)** of metastatic nodules in lung. **p < 0.01. Scale bars, 2mm and 250μm. **(F)** H&E and immunohistochemical staining images of NSCLC cancer tissues, PDX and derived PDO. Scale bars, 50 µm. **(G)** To evaluate heterogeneous expression of BGA5 in tumor, the constructed PDOs were separated with a filter with 100μm pore size and BAG5 expression was investigated using histochemical staining. **(H)** The PDO (PDO#1) with higher basal BAG5 expression were infected with gRNA guided BAG5 using CRISPR/Cas9 system for knockout. Western blot analysis confirming effective BAG5 knockout in PDO#1. β-actin was used as a loading control. **(I)** Control and BAG5 knockout PDOs were seeded and maintained for 1 week. Representative photographs (left) of colony formation and their quantitative analysis (right) were presented. Data represent the mean ± SD of three independent experiments. Statistical significance was assessed by unpaired two-tailed Student’s t-test; **p < 0.001. **(J)** Invasion of cells to the lower compartment and chemotaxis of the upper compartment organoids was evaluated by Matrigel-uncoated Transwell. Representative photographs (left) and their numbers (right). These data show mean ± SD of three independent experiments. *p < 0.05.

Given the strong association of BAG5 with tumor progression, we next evaluated its functional role in NSCLC using genetic knockout strategies. BAG5 knockout inhibited proliferation, colony formation, migration and invasion, spheroid formation, and colony formation on soft agar of NSCLC cells *in vitro* (**Supplementary Figure S1**). *In vivo*, BAG5 knockout led to marked reductions in tumor weight, volume, and Ki67 expression in xenograft mouse models (**Figures 3A–C**). Additionally, lung metastatic nodules were significantly reduced (**Figures 3D, E**), indicating BAG5’s role in promoting metastatic dissemination.

To validate these findings in clinically relevant models, we generated PDX and PDO systems from patient-derived tumor tissues. These models preserved native tumor architecture and BAG5 expression patterns (**Figure 3F**). Organoids with higher BAG5 expression displayed greater growth capacity and invasive potential, which were suppressed by BAG5-targeting sgRNAs (**Figures 3G–J**).

Together, these *in vitro* and *in vivo* data confirm that BAG5 promotes NSCLC cell proliferation and metastasis, reinforcing its role as a functional oncogene.

### 3.3 Enrichment of hallmark genes involved in epithelial mesenchymal transition and metabolic reprogramming in BAG5^+^ NSCLC tumor cells

**Figure 4:**
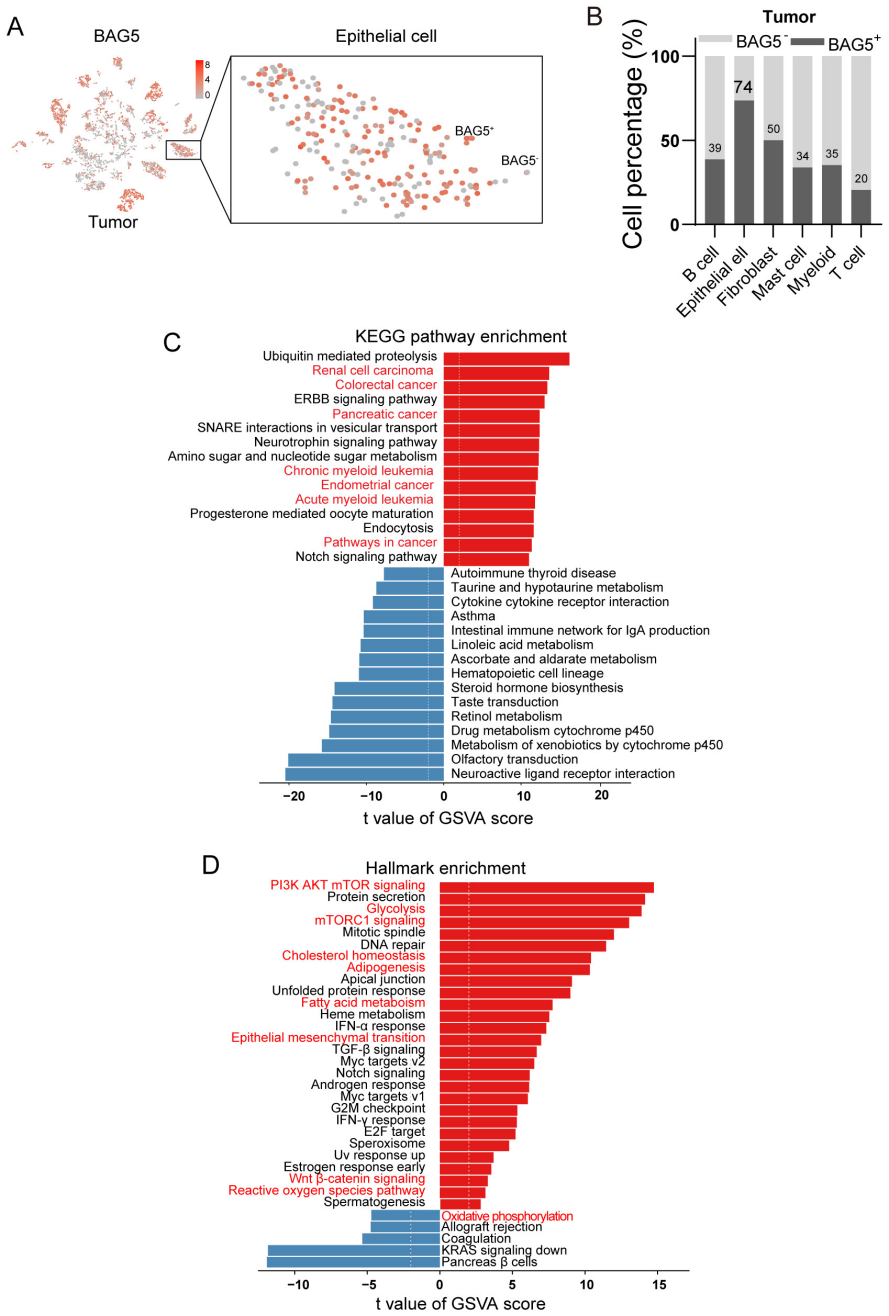
Enrichment of hallmark genes involved in EMT and metabolic reprogramming in BAG5^+^ NSCLC tumor cells. **(A)** The t-SNE plot of BAG5 expression landscape in NSCLC tumor tissues. Heterogeneity in the expression of BAG5 was shown in tumor epithelial cells. **(B)** The relative proportion of BAG5^+^ cells from different cell types in NSCLC tissues. **(C, D)** Single sample GSVA (ssGSVA) analysis (BAG5^+^ vs. BAG5^-^ tumor epithelial cells) showing most enriched KEGG pathways **(C)** and Hallmark annotation **(D)**.

To investigate the cellular functions enriched in BAG5+ tumor cells, we analyzed single-cell transcriptomic data from NSCLC epithelial cells. Despite high overall expression (74%), BAG5 exhibited heterogeneous distribution across epithelial subpopulations (**Figures 4A, B**). Gene set variation analysis (GSVA) revealed that BAG5+ cells were enriched in multiple oncogenic pathways, including those involved in colorectal, pancreatic, and endometrial cancers (**Figure 4C**), which suggests that BAG5 may converge on evolutionarily conserved oncogenic programs. These tumor types share molecular features such as EMT, metabolic reprogramming, and cell cycle deregulation, which is consistent with the phenotypes observed following BAG5 modulation in our NSCLC models.

Hallmark gene sets showed strong enrichment for metabolic reprogramming (cholesterol homeostasis, adipogenesis, fatty acid metabolism, reactive oxygen species pathway, oxidative phosphorylation), metabolic stress pathways (PI3K/AKT/mTOR signaling, mTORC1 signaling), Wnt β-catenin signaling and epithelial mesenchymal transition (EMT) (**Figure 4D**).

These findings indicate that BAG5+ tumor epithelial cells are characterized by enhanced EMT and metabolic reprogramming, supporting their role in aggressive tumor phenotypes.

### 3.4 Involvement of BAG5 interactome in regulation of actin cytoskeleton, focal adhesion and RNA metabolism

To elucidate BAG5’s molecular functions, we performed immunoprecipitation (IP) followed by mass spectrometry to identify interacting proteins in NSCLC versus normal epithelial cells. BAG5 formed distinct protein complexes in A549 cells (**Figure 1A**), including known chaperone partners such as HSPA8 (**Figure 1B**) (5) (13). A total of 130 proteins were uniquely associated with BAG5 in NSCLC cells (**Figure 1C**, **Supplementary Table S1**).

GO and KEGG analyses revealed that these interactors were involved in actin cytoskeleton regulation, cell motility, and focal adhesion (**Figures 1D, E**),which are closely related with migratory and invasive abilities of cells. ClueGO network analysis further showed that 25% of interactors were enriched in heat stress responses (**Figure 1F**). Top interactors included HSPA8, DHX9, and IGF2BP1-3, all implicated in RNA metabolism (**Figure 1G**), and co-IP confirmed these interactions (**Figure 1H**). Notably, BAG5 knockout did not alter the expression of these partners (**Figure 1I**).

These results suggest that BAG5 contributes to tumor progression by orchestrating protein complexes involved in cytoskeletal remodeling, cell adhesion, and RNA processing.

### 3.5 Enrichment of BAG5-regulated proteome in focal adhesion and EMT

**Figure 5:**
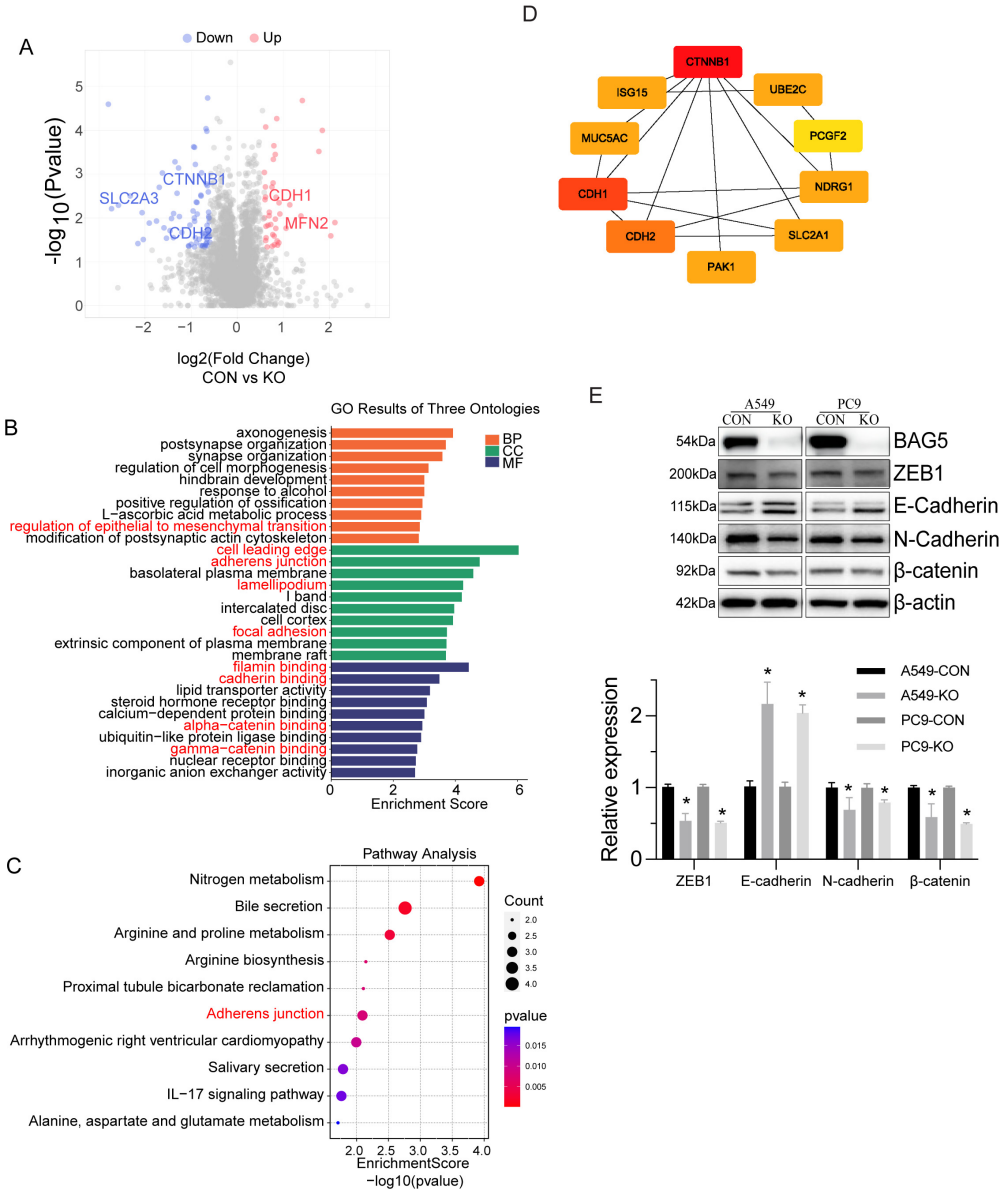
Enrichment of BAG5-regulated proteome in focal adhesion and EMT. **(A)** Volcano plots of DEGs from quantitative proteomics by comparing control and BAG5 knockout A549 cells. Differential gene expression was defined by *p* value< 0.05 and a log_2_FC>|0.6|. **(B, C)** GO **(B)** and KEGG pathways **(C)** enrichment analysis of DEGs. **(D)** Key hub molecules of BAG5-regualted proteome involving EMT were visualized by Cytoscape. **(E)** Western blot analysis was performed to confirm BAG5-regulatory EMT hub molecules. *p < 0.05.

To further delineate BAG5-mediated downstream effects, we conducted quantitative proteomic profiling upon BAG5 knockout. A total of 99 proteins were differentially expressed (44 downregulated, 55 upregulated) (**Figure 5A**, **Supplementary Table S2**). Consistent with single cell transcriptome (**Figure 4D**) and interactome data (**Figures 1D, E**), enrichment analysis highlighted pathways in EMT, focal adhesion, and cytoskeleton regulation (**Figures 5B, C**).

Network visualization by Cytoscape identified key EMT-related proteins including CTNNB1, CDH1, and CDH2 (**Figure 5D**). Western blot validation showed that BAG5 knockout increased E-cadherin while decreasing N-cadherin, ZEB1, and β-catenin expression (**Figure 5E**).

Among the key EMT-associated nodes identified, CTNNB1 (β-catenin) plays a central role in canonical Wnt signaling and is known to drive stemness, therapeutic resistance, and tumor aggressiveness in NSCLC (25). Equally, the cadherin switch—downregulation of CDH1 (E-cadherin) and upregulation of CDH2 (N-cadherin)—is a hallmark of EMT that enhances migratory and invasive behavior across cancer types, including NSCLC (26). These convergent molecular changes underscore BAG5’s influence on EMT reprogramming and metastatic potential.

These data support a role for BAG5 in regulating the EMT phenotype and cytoskeletal organization in NSCLC cells.

### 3.6 Enrichment of phosphorproteome affected by BAG5 in RNA metabolism, focal adhesion and cytoskeleton

**Figure 6:**
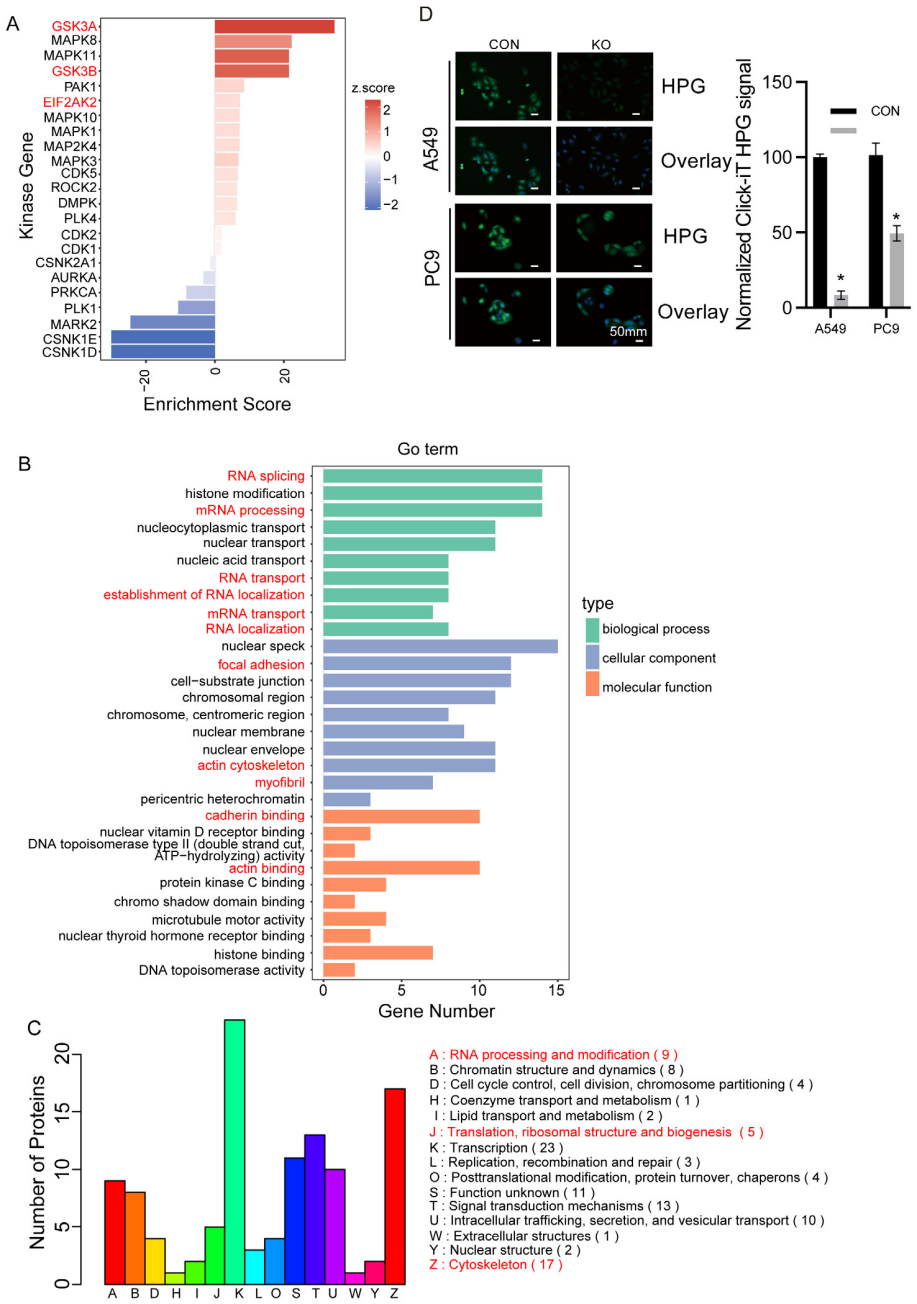
Enrichment of phosphorproteome affected by BAG5 in RNA metabolism, focal adhesion and cytoskeleton. **(A)** KSEA (kinase set enrichment assay) of quantitative phosphorproteomic data. **(B, C)** GO **(B)** and COG **(C)** enrichment analysis of DEGs. **(D)** HPG incorporation assay for nascent protein synthesis analysis. Quantified fluorescence intensity of HPG signal is shown at the bottom (mean ± SD, n = 3, *p < 0.05, Student’s t-test). Scale bars, 50 μm.

To investigate BAG5’s effect on phosphorylation networks, we performed phosphoproteomic analysis. Kinase set enrichment analysis (KSEA) showed activation of GSK3A/B and EIF2αK2 upon BAG5 knockout (**Figure 6A**, **Supplementary Table S3**). This aligns with PI3K/AKT/mTOR pathway suppression observed in BAG5+ cells (**Figure 4D**).

GO and COG enrichment of BAG5-regulated phosphoproteins revealed roles in RNA metabolism, translation, focal adhesion, and cytoskeletal regulation (**Figures 6B, C**).

Interestingly, among the enriched pathways in the phosphoproteomic dataset, transcription-related processes displayed the highest number of differentially phosphorylated proteins (**Figure 6C**), indicating that BAG5 may influence transcriptional regulation indirectly through phosphorylation-mediated signaling cascades.

In addition, eIF2αK2, a kinase to suppress translation globally via phosphorylation of eIF2α, was activated by BAG5 knockout (**Figure 6A**). HPG incorporation was then performed to investigate the potential involvement of BAG5 in global protein synthesis. Overall nascent protein synthesis was significantly reduced by BAG5 knockdown in both A549 and PC9 cells (**Figure 6D**).

These results indicate that BAG5 maintains translation capacity and supports motility-related signaling via modulation of the phosphoproteome.

### 3.7 Regulation of metabolic reprogramming by BAG5 in NSCLC

**Figure 7:**
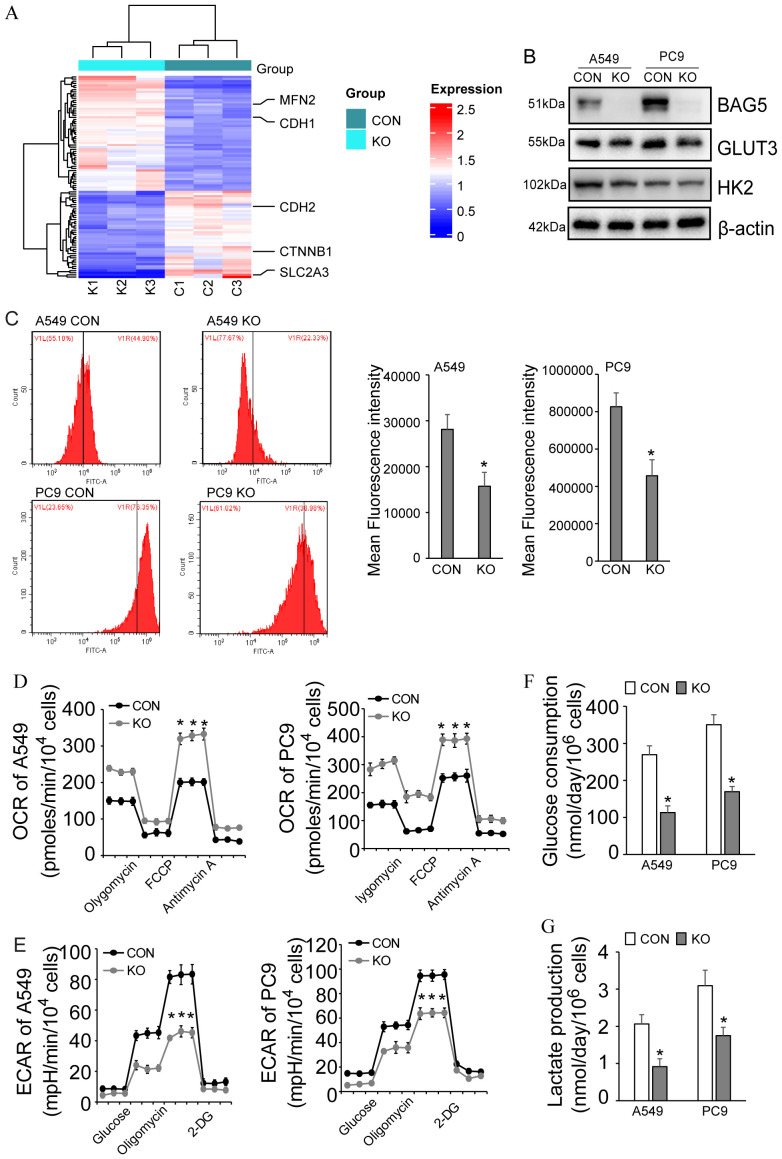
Regulation of metabolic reprogramming by BAG5 in NSCLC. **(A)** Heat map of differentially expressed proteins including metabolic reprogramming, EMT and mitodynamics-related ones in CON and BAG5-KD A549 cells by quantitative proteomics. **(B)** Downregulation of GLUT3 by BAG5 knockout was confirmed by western blot analysis in NSCLC cells. **(C)** Glucose uptake by NSCLC cells was analyzed by 2-NBDG incorporation experiments. **(D, E)** OCR **(D)** and ECAR **(E)** were measured using seahorse instrument in control and BAG5 knockout A549 and PC9 cells. **(F, G)** Glucose consumption **(F)** and lactate production **(G)** was analyzed by spectrophotometric methods. Data represent mean ± SD; *p < 0.05.

To functionally validate BAG5’s role in metabolic reprogramming (**Figure 4D**), we examined its impact on glucose metabolism. BAG5 knockout reduced protein and mRNA levels of GLUT3 (SLC2A3), the member with the highest glucose affinity among the glucose transporters (**Figures 5A**, **7A, B**), which was not reflected at the mRNA level, indicating post-transcriptional regulation.

Consistent with GLUT3 suppression, BAG5 knockout reduced glucose uptake (2-NBDG assay), glucose consumption and lactate production, decreased ECAR, and elevated OCR, indicative of a shift away from glycolysis toward oxidative metabolism (**Figures 7C–G**).

These results demonstrate that BAG5 supports glycolytic metabolism in NSCLC, primarily through post-transcriptional regulation of GLUT3.

### 3.8 Implication of BAG5 in mitochondrial dynamics of NSCLC

**Figure 8:**
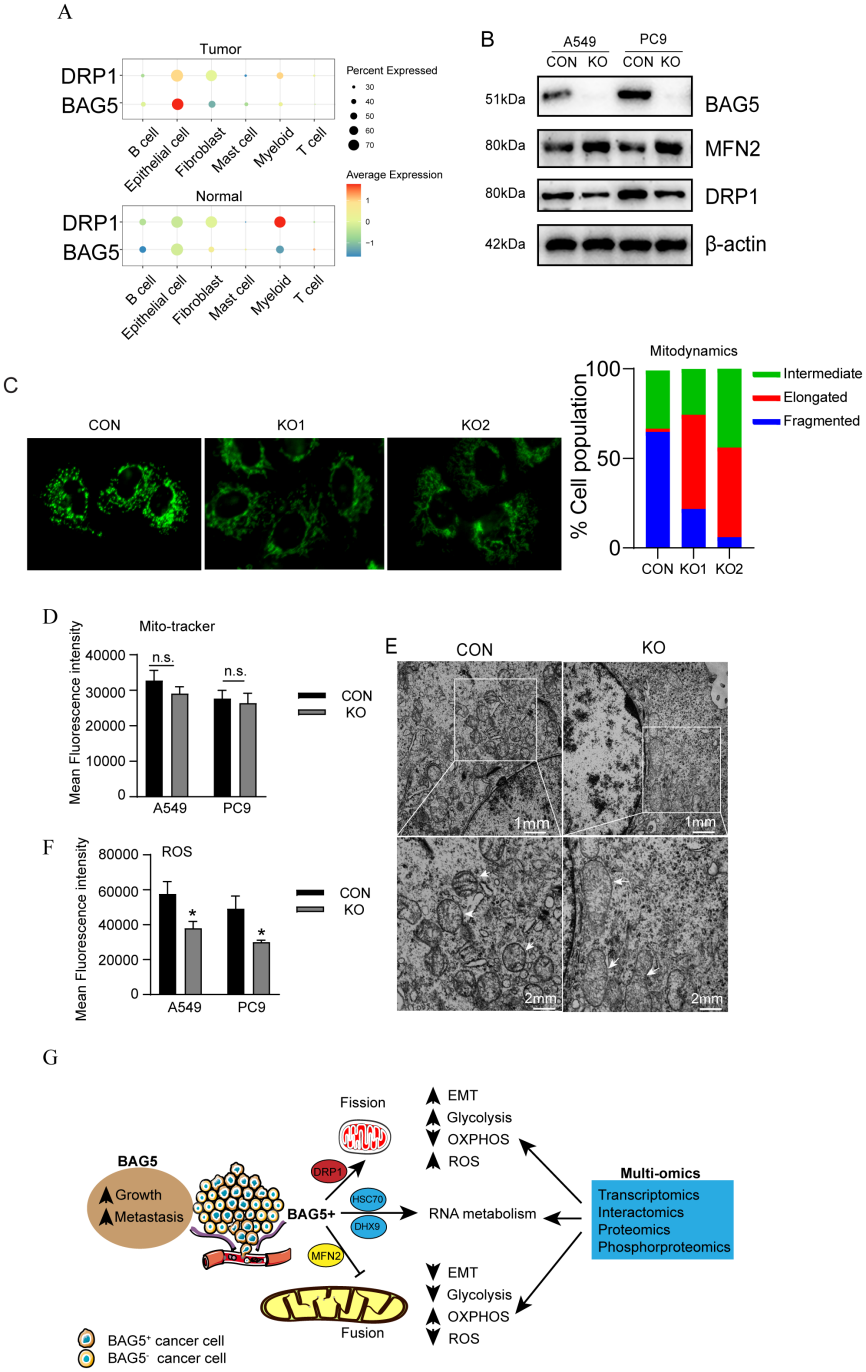
Regulation of mitochondrial dynamics by BAG5 in NSCLC. **(A)** co-expression of BAG5 and DRP1 transcripts was explored using the single cell transcriptome. **(B)** Regulation of NFN2 and DRP1 by BAG5 in NSCLC cells was investigated via western blot analysis. **(C)** Representative images of the mitochondrial morphological change by mitotracker staining in A549 cells with control or BAG5 knockout (left). The proportion of cells (n = 100 cells for each sample) with fragmented, intermediate and elongated mitochondria was quantifified (right).Scale bars, 1 μm or 2 μm. **(D)** MFI of mitotracker staining was measured using flow cytometry in control or BAG5 knockout NCSCLs. **(E)** Representative TEM images of A549 cells confirmed the mitochondrial morphological changes by BAG5 downregulation. **(F)** Cells with control or BAG5 knockout were incubated with 10 μM DCFH-DA for 30 min. The MFI of intracellular ROS level was measured with flow cytometry. Data represent mean ± SD; *p < 0.05; n.s. means no significance (Student’s t test). **(G)** A proposed model for function and mechanism of BAG5 in NSCLC. Based on the multi-omics data, BAG5, as an oncogene, was shown to affect the malignant phenotype of NSCLC cancer cells, and participate in multiple tumor pathways, including regulation of mitochondrial morphology, metabolic reprogramming, RNA metabolism, cytoskeleton, and EMT, which consequently promoting tumor growth and metastasis.

Given BAG5’s impact on metabolism and oxidative stress, we explored its role in mitochondrial dynamics. The proteomic results found that BAG5 knockout in NSCLC cells significantly increased MFN2, the gene involved in fusion of mitochondria (**Figures 5A**, **7A**). In addition, single cell transcriptome revealed the high co-expression of BAG5 and DRP1, the gene involved in fission of mitochondria, in NSCLC epithelial tumor cells (**Figure 8A**). Western blotting confirmed upregulation of MFN2 while downregulation of DRP1 by BAG5 knockout in NSCLC (**Figure 8B**).

In addition, consistent with activation of ROS pathway in BAG5^+^ cluster (**Figure 4D**), flow cytometry demonstrated that knockout of BAG5 significantly inhibited ROS production in A549 and PC9 cells (**Figure 8F**).

Consistent with increased MFN2 and reduced DRP1 expression, mitotracker staining demonstrated that downregulation of BAG5 significantly promoted mitochondrial fusion, leading to elongation and excessive fusion of mitochondria (**Figure 8C**), but did not significantly affect mitochondrial mass as measured by MitoTracker fluorescence intensity (**Figure 8D**), indicating that BAG5 regulates mitochondrial morphology rather than abundance. The effect of BAG5 downregulation on mitochondrial morphological changes was further confirmed by transmission electron microscopy (TEM) (**Figure 8E**). Additionally, consistent with activation of ROS pathway in BAG5^+^ cluster (**Figure 4D**), flow cytometry showed a significant reduction in ROS levels (**Figure 8F**).

These findings suggest that BAG5 promotes mitochondrial fission and ROS production, further supporting its role in metabolic and oxidative reprogramming of NSCLC cells.

## 4 Discussion

NSCLC is the most lethal disease in malignancy, and conventional surgery and platinum-based combination chemotherapy are currently the most commonly used therapies in the clinic (27). With the rapid development of molecular biology, molecularly targeted therapies, as well as immunotherapy, have become first-line treatment options (2) that have substantially improved outcomes in some patients, but overall five-year survival remains less than 15% (27–30). Therefore, it is urgent to use advanced techniques and models to explore novel biomarkers and molecular mechanisms to improve the prognosis of patients.

BAG5 is a special member of the BAG family of proteins and consists of five BAG domains (3). Several lines of evidence has indicated that BAG5 may promote or suppress progression of tumors in a context-dependent manner. At present, the function of BAG5 in NSCLC remains unknown. The current study found that BAG5 knockout suppressed the malignant phenotype of NSCLC cell lines, including proliferation, migration and stem cell phenotype. At present, patient-derived tumor xenograft (PDX) and patient-derived organoid (PDO) have been established as preclinical models for translational research, since both PDX and PDO can maintain the histopathological structure and gene expression profile of the original tumors (31–33). The current study also used PDX and PDO preclinical models to further verify that BAG5 knockout indeed significantly suppressed the proliferation and invasion of NSCLC, emphasizing it as a potential molecular target for NSCLC intervention.

The BAG protein family comprises multifunctional proteins that interact with a variety of proteins and is involved in various cellular processes, such as stress signaling, cell cycle and tumorigenesis (3, 4, 34). Through the integrative analysis of high-throughput molecular “multi-omics” data sets (from bulk transcriptome, single cell transcriptome to proteome, interactome and phosphoproteome), the current study delineated a multi-dimensional molecular map, and explored the molecular changes and pathways regulated by BAG5 in NSCLC on this map. With the development of single cell sequencing technology, it has become possible to analyze the molecular characteristics of lung cancer at the single cell level (35–37). By reanalyzing public scRNA-Seq data sets (GSE119911), the current study identified heterogeneous expression of BAG5 in NSCLC tumor epithelial cells, which rationally explain the discrepancy between increased BAG5 expression and its lack of prognostic significance in the TCGA cohort (38). Based on the multi-omics data and functional analysis, the current study demonstrated that BAG5 was involved in a variety of tumor pathways, including metabolic reprogramming, mitochondria dynamics, metabolism of RNA and EMT, to facilitate proliferation and invasion of NSCLC. Compared with one-dimensional molecular omics data, the current integrative research of multiple molecular omics data sets provided more comprehensive and in-depth insights into function of BAG5 in NSCLC.

Metabolic reprogramming of glucose, glutamine and ketone bodies, especially aerobic glycolysis is considered as the basic feature of tumor metabolic disorder, not just the result of passive response to mitochondrial damage (39). This metabolic reprogramming promotes tumor survival to meeting the cellular demands for energy and macromolecule synthesis (40). Targeted metabolic reprogramming may be a potential therapeutic strategy for cancers such as NSCLC (41, 42). Glucose transport mediated by glucose transporter (GLUT) family is critical for tumor cell metabolism and functions as the pacesetter of aerobic glycolysis. Among the four glucose transporters, GLUT3 has the highest affinity with glucose. Based on the multi-omics data, the current study identified that GLUT3 was particularly regulated by BAG5 in NSCLC. Several studies have reported that GLUT3 is induced at the transcriptional level to promote aerobic glycolysis of tumors. For example, GLUT3 is transactivated by Ying Yang 1 (YY1) and promotes the Warburg effect of colon carcinoma (43). Transient receptor potential melastatin 7 (TRPM7) functions as a gatekeeper of cellular glycolysis in glycolytic cancer and endothelial cells via transcriptional regulation of GLUT3 (44). GLUT3 is also transcriptionally activated by NFκB and is critical for oncogenic mTORC1-mediated aerobic glycolysis and tumorigenesis (45). In addition, HMGA1-mediated transcriptional activation of GLUT3 is responsible for oncogenic functions of caveolin 1 in colorectal cancer (46, 47). Different from the transcriptional regulation of GLUT3 by various oncogenic factors, the current study demonstrated that BAG5 knockout decreased GLUT3 protein expression, while unaltered its mRNA expression, indicating that BAG5 might regulate GLUT3 at the protein level. GLUT3 was not included in the interactome of BAG5, molecular docking also confirmed that BAG5 could not directly interacted with GLUT3 (data not shown). Since BAG5 affected global protein translation possibly via interaction with proteins implicated in RNA metabolism or/and eIF2α phosphorylation, BAG5 might affect GLUT3 expression at the translational level. The exact mechanism by which BAG5 regulated GLUT3 protein expression in NSCLC requires further investigation.One plausible explanation is that BAG5 may influence GLUT3 expression via translational regulation rather than direct protein interaction. Our BAG5 interactome data identified multiple RNA-binding proteins, including IGF2BP1–3, which are well-known regulators of mRNA stability and translation of oncogenic transcripts such as MYC and GLUT family members. Additionally, our phosphoproteomic analysis revealed increased phosphorylation of eIF2α and activation of its upstream kinase EIF2AK2 following BAG5 knockout, which are indicative of translational repression. These findings suggest that BAG5 may act as a scaffold protein to stabilize translation-promoting complexes or prevent stress-induced translational shutdown, thereby sustaining GLUT3 protein levels. Although further studies are required to confirm this mechanism, the evidence supports a role for BAG5 in post-transcriptional modulation of metabolic regulators through translational control machinery.

Metabolic reprogramming of tumor cells is closely related to the changes of mitochondrial dynamics (48, 49). Mitochondrial dynamics is mainly divided into fusion and division, which is the key mechanism for regulating cell redox state, organelle function and cell death (50). Tumor cells maintain their proliferation and survival by altering the mitochondrial kinetic homeostasis (51). Mitochondrial division has an oncogenic effect (52), while mitochondrial fusion suppresses tumor progression (53). Inhibition of mitochondria division inhibits cell proliferation, epithelial-mesenchymal transformation and metastasis of various cancer cells (54, 55). Consistent with these literatures, the current study demonstrated that BAG5 knockout suppressed proliferation and invasion of NSCLC, simultaneously resulted in increased mitochondrial fusion, as evidenced by BAG5 knockout increased mitochondria with elongated morphology, as well as increased mitochondrial fusion gene MFN2 expression, while decreased mitochondrial fission gene DRP1expression.

Importantly, although BAG5 knockdown did not significantly change total mitochondrial mass, as reflected by unaltered MitoTracker fluorescence intensity (MFI), it led to markedly elevated oxygen consumption rate (OCR), suggesting enhanced oxidative phosphorylation (OXPHOS) efficiency. This apparent discrepancy can be explained by mitochondrial morphological remodeling. Mitochondrial fusion, promoted by BAG5 knockout, is known to increase cristae surface area and respiratory capacity per mitochondrion, thereby improving bioenergetic efficiency without increasing mitochondrial number or mass. These findings support the conclusion that BAG5 regulates mitochondrial function not by altering organelle quantity, but through modulating mitochondrial dynamics and morphology to favor metabolic reprogramming.

Although our flow cytometry results confirmed that BAG5 knockout significantly decreased ROS levels, we acknowledge that a more comprehensive characterization of oxidative stress, such as analysis of GSH : GSSG ratios or expression of antioxidant response genes (e.g., SOD2, GPX4, NRF2), would further strengthen the mechanistic insight into BAG5-mediated redox regulation. Previous studies have shown that increased mitochondrial fission promotes ROS accumulation and tumor aggressiveness, while enhanced mitochondrial fusion reduces ROS generation and suppresses metastasis (56–59). Our findings are consistent with these observations and suggest that BAG5 may exert its pro-tumorigenic effects, at least in part, through maintaining mitochondrial fission and oxidative stress. Future studies using redox-sensitive reporter systems or targeted metabolomic profiling would be valuable to delineate this mechanism more precisely.

This study has certain limitations. While PDO and xenograft models were employed, the *in vivo* validation of pathway-specific BAG5 inhibition remains to be conducted. Additionally, BAG5 expression and function may vary across NSCLC subtypes and genetic backgrounds, which warrants further investigation. Future studies should aim to identify small-molecule inhibitors or peptides that disrupt BAG5 interaction with its key partners, particularly RNA-binding proteins involved in metabolic regulation. Elucidating the context-specific dependencies of BAG5 will help refine its therapeutic potential. In adition, although our findings suggest that reduced GLUT3 protein levels contribute to the suppressed glycolysis and proliferation in BAG5-KO cells, the absence of a GLUT3 rescue experiment remains a limitation. Future studies will be directed at validating this mechanism through targeted rescue of GLUT3 in BAG5-depleted models.

Our integrative multi-omics framework—spanning single-cell RNA sequencing, quantitative proteomics, and phosphoproteomic mapping—enabled a high-resolution dissection of BAG5-related molecular networks. This systems-level approach revealed converging pathways in metabolism, cytoskeleton remodeling, and EMT, providing a roadmap for rational therapeutic targeting in BAG5-high NSCLC cases.

To sum up (**Figure 8G**), among the multiple pathways modulated by BAG5, our data suggest that post-transcriptional regulation of glucose metabolism via GLUT3, and modulation of mitochondrial dynamics, represent the most promising therapeutic axes. Specifically, BAG5 knockout reduced GLUT3 protein levels without altering mRNA expression, indicating a possible translational regulatory mechanism. Given that GLUT3 is essential for aerobic glycolysis and tumor cell proliferation, this axis could be selectively exploited, especially in tumors with high BAG5 and GLUT3 co-expression. Furthermore, our proteomic interactome suggests BAG5 may regulate translation through interaction with RNA-binding proteins such as IGF2BP1–3 and factors associated with EIF2α phosphorylation, which have been implicated in cancer cell metabolic control.

In parallel, BAG5 also modulates mitochondrial fission-fusion balance, as evidenced by increased MFN2 and decreased DRP1 expression upon BAG5 silencing, leading to enhanced mitochondrial fusion and reduced tumor aggressiveness. Notably, excessive mitochondrial fission is increasingly recognized as a hallmark of invasive tumors, and several DRP1 inhibitors (e.g., Mdivi-1) have shown therapeutic potential. Thus, targeting BAG5 may indirectly impact this pathway and synergize with existing mitochondrial-targeting therapies.

Taken together, these findings suggest that while BAG5 regulates multiple oncogenic processes, its most druggable and central effects lie in translational control of metabolic effectors and mitochondrial dynamics. These dual functions position BAG5 as a potential node regulator, offering opportunities for precision intervention in BAG5-high NSCLC subtypes. Future efforts should focus on mechanistic dissection of its protein interaction network and high-throughput screening for small molecules that disrupt BAG5-RNA-binding protein complexes or modulate its effect on mitochondrial regulators.

## Data Availability

The omics data in this article are either publicly available or provided as supplementary files. Public scRNA-Seq data sets are available from Gene Expression Omnibus (GEO) (GSE119911). Interactome proteomics and phosphoproteomics data are available within the supplementary files. No other data is restricted.
