# Supplementary figures and images for: Mechanistic insights into promotion of non-small cell lung cancer by BAG5 using integrative multi-omics approaches
Source: Front Immunol. 2025 Jul 25;16:1648139. doi: 10.3389/fimmu.2025.1648139 (PMC12331601; doi:10.3389/fimmu.2025.1648139)

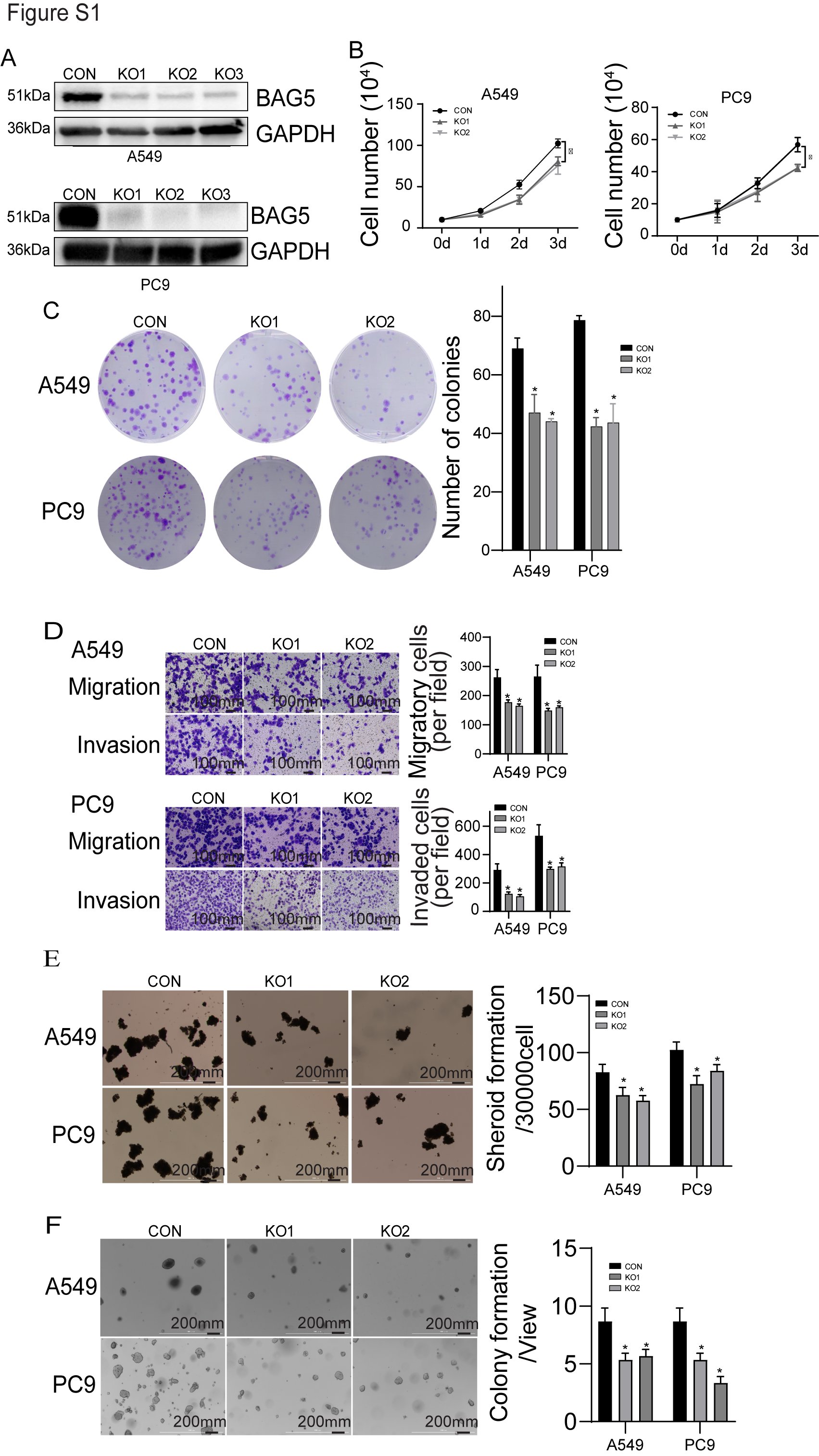

Supplement: Supplementary file 4 [file Image1.tif]
