# Supplementary material for: Mechanistic insights into promotion of non-small cell lung cancer by BAG5 using integrative multi-omics approaches
Source: Front Immunol. 2025 Jul 25;16:1648139. doi: 10.3389/fimmu.2025.1648139 (PMC12331601; doi:10.3389/fimmu.2025.1648139)
Supplement: Supplementary file 5 [file DataSheet1.docx]

| BGA5 | | | | |  |
| --- | --- | --- | --- | --- | --- |
| Dataset | *P*value | HR | 95%CI | Prognostic |  |
| GSE13213 | 0.0023 | 2.4595 | 1.3795 ~ 4.3849 | Poor |  |
| Stage II | 0.8006 | 0.7579 | 0.0881 ~ 6.5165 |  |  |
| Stage III | 0.5659 | 1.3558 | 0.4795 ~ 3.8337 |  |  |
| GSE30219 | 0.0056 | 1.5448 | 1.1354 ~ 2.1017 | Poor |  |
| GSE31210 | 0.0363 | 2.0607 | 1.0473 ~ 4.0546 | Poor |  |
| Stage I | 0.0602 | 2.4927 | 0.9616 ~ 6.4620 |  |  |
| Stage II | 0.2424 | 1.7967 | 0.6727 ~ 4.7992 |  |  |
| GSE5843 | 0.1037 | 2.0460 | 0.8639 ~ 4.8456 |  |  |
| Stage I | 0.1245 | 2.0424 | 0.8213 ~ 5.0795 |  |  |
| GSE67639 | 0.1615 | 1.1642 | 0.9410 ~ 1.4404 |  |  |
| GSE11117 | 0.2017 | 1.7867 | 0.7331 ~ 4.3544 |  |  |
| Stage IV | 0.0858 | 2.9187 | 0.8598 ~ 9.9078 |  |  |
| GSE12428 | 0.2125 | 1.9878 | 0.6750 ~ 5.8543 |  |  |
| GSE29066 | 0.2183 | 1.4780 | 0.7935 ~ 2.7531 |  |  |
| Stage I | 0.9247 | 1.0392 | 0.4683 ~ 2.3060 |  |  |
| Stage II | 0.0320 | 4.1116 | 1.1292 ~ 14.9713 | Poor |  |
| GSE33072 | 0.2218 | 0.6812 | 0.3680 ~ 1.2611 |  |  |
| GSE26939 | 0.2719 | 0.7286 | 0.4141 ~ 1.2818 |  |  |
| Stage I | 0.2320 | 0.5969 | 0.2562 ~ 1.3912 |  |  |
| Stage II | 0.4152 | 1.5969 | 0.5179 ~ 4.9237 |  |  |
| Stage III | 0.5568 | 0.6288 | 0.1338 ~ 2.9551 |  |  |
| GSE19188 | 0.2774 | 0.6808 | 0.3402 ~ 1.3625 |  |  |
| GSE102287 | 0.2948 | 1.6928 | 0.6324 ~ 4.5313 |  |  |
| GSE4573 | 0.3042 | 1.3163 | 0.7792 ~ 2.2234 |  |  |
| Stage I | 0.1238 | 1.7742 | 0.8548 ~ 3.6827 |  |  |
| Stage II | 0.1748 | 2.1181 | 0.7164 ~ 6.2629 |  |  |
| Stage III | 0.5312 | 0.6919 | 0.2185 ~ 2.1912 |  |  |
| GSE5123 | 0.3384 | 0.6201 | 0.2331 ~ 1.6496 |  |  |
| Stage I | 0.4211 | 0.5385 | 0.1192 ~ 2.4324 |  |  |
| Stage II | 0.5547 | 0.6216 | 0.1283 ~ 3.0107 |  |  |
| Stage III | 0.7106 | 0.6507 | 0.0672 ~ 6.2998 |  |  |
| GSE3141 | 0.3597 | 0.7358 | 0.3816 ~ 1.4188 |  |  |
| GSE14814 | 0.4219 | 0.7772 | 0.4202 ~ 1.4377 |  |  |
| Stage I | 0.8684 | 0.9261 | 0.3734 ~ 2.2966 |  |  |
| Stage II | 0.6200 | 0.8170 | 0.3675 ~ 1.8162 |  |  |
| GSE37745 | 0.4603 | 1.1504 | 0.7931 ~ 1.6688 |  |  |
| Stage I | 0.1051 | 1.4631 | 0.9234 ~ 2.3184 |  |  |
| Stage II | 0.5404 | 0.7520 | 0.3019 ~ 1.8728 |  |  |
| Stage III | 0.1314 | 2.0424 | 0.8077 ~ 5.1650 |  |  |
| GSE17710 | 0.4629 | 1.3270 | 0.6234 ~ 2.8248 |  |  |
| Stage I | 0.1213 | 2.2091 | 0.8105 ~ 6.0213 |  |  |
| Stage II | 0.9591 | 1.0323 | 0.3068 ~ 3.4732 |  |  |
| GSE31908_GPL97 | 0.4904 | 0.5637 | 0.1106 ~ 2.8744 |  |  |
| GSE11969 | 0.5258 | 0.8353 | 0.4790 ~ 1.4565 |  |  |
| Stage I | 0.0590 | 0.3121 | 0.0932 ~ 1.0451 |  |  |
| Stage II | 0.7086 | 0.7482 | 0.1634 ~ 3.4262 |  |  |
| Stage III | 0.7577 | 1.1264 | 0.5289 ~ 2.3985 |  |  |
| Roepman | 0.5588 | 0.8228 | 0.4279 ~ 1.5822 |  |  |
| GSE50081 | 0.6508 | 0.8847 | 0.5206 ~ 1.5035 |  |  |
| Stage I | 0.6167 | 1.1723 | 0.6290 ~ 2.1851 |  |  |
| Stage II | 0.2727 | 0.5800 | 0.2191 ~ 1.5352 |  |  |
| GSE31908_GPL96 | 0.6587 | 0.7015 | 0.1455 ~ 3.3827 |  |  |
| GSE10245 | 0.7595 | 0.8441 | 0.2851 ~ 2.4992 |  |  |
| GSE29013 | 0.7600 | 1.1750 | 0.4175 ~ 3.3074 |  |  |
| Stage I | 0.2679 | 2.8008 | 0.4530 ~ 17.3182 |  |  |
| Stage III | 0.3999 | 1.8607 | 0.4383 ~ 7.8988 |  |  |
| GSE41271 | 0.7921 | 1.0558 | 0.7051 ~ 1.5810 |  |  |
| Stage I | 0.9110 | 0.9597 | 0.4662 ~ 1.9754 |  |  |
| Stage II | 0.2593 | 0.4956 | 0.1463 ~ 1.6782 |  |  |
| Stage III | 0.1698 | 1.4918 | 0.8428 ~ 2.6406 |  |  |
| GSE68465 | 0.8042 | 1.0380 | 0.7728 ~ 1.3942 |  |  |
| TCGA | 0.9210 | 0.9885 | 0.7870 ~ 1.2416 |  |  |
| Combined | 0.0605 | 1.0945 | 0.9959 ~ 1.2029 |  |  |

Note: cutoff: upper 25% vs other 75%
